# Entropy of Iterated Function Systems and Their Relations with Black Holes and Bohr-Like Black Holes Entropies

**DOI:** 10.3390/e20010056

**Published:** 2018-01-12

**Authors:** Christian Corda, Mehdi FatehiNia, MohammadReza Molaei, Yamin Sayyari

**Affiliations:** 1 Research Institute for Astronomy and Astrophysics of Maragha (RIAAM), Maragha 82641, Iran; 2 International Institute for Applicable Mathematics & Information Sciences (IIAMIS), B.M. Birla Science Centre, Hyderabad 500 463, India; 3 Mahani Mathematical Research Center, Shahid Bahonar University of Kerman, Kerman 93630, Iran; 4 Department of Mathematics, Yazd University, Yazd 89195-741, Iran

**Keywords:** iterated function system, Axiom *A*, metric entropy, topological entropy, black hole entropy, Bohr-like black hole

## Abstract

In this paper we consider the metric entropies of the maps of an iterated function system deduced from a black hole which are known the Bekenstein–Hawking entropies and its subleading corrections. More precisely, we consider the recent model of a Bohr-like black hole that has been recently analysed in some papers in the literature, obtaining the intriguing result that the metric entropies of a black hole are created by the metric entropies of the functions, created by the black hole principal quantum numbers, i.e., by the black hole quantum levels. We present a new type of topological entropy for general iterated function systems based on a new kind of the inverse of covers. Then the notion of metric entropy for an Iterated Function System (IFS) is considered, and we prove that these definitions for topological entropy of *IFS’s* are equivalent. It is shown that this kind of topological entropy keeps some properties which are hold by the classic definition of topological entropy for a continuous map. We also consider *average entropy* as another type of topological entropy for an IFS which is based on the topological entropies of its elements and it is also an invariant object under topological conjugacy. The relation between Axiom *A* and the average entropy is investigated.

## 1. Introduction

This article begins with the quantum black hole (BH) physics. Referring to the recent Bohr-like BH model [1,2,3], we see that the Bekenstein–Hawking entropy and its subleading corrections is a metric entropy of an iterated function system, and we see that the metric entropy of a BH is function of the BH principal quantum number (the “overtone” number). We know that the topological entropy is an invariant object under topological conjugate relation which denotes the measure of the complexity of a dynamical system. Topological entropy for a continuous map f:X→X on a compact metric space (X,d) has been considered from different viewpoints [4,5,6,7,8]. In [4], the authors introduce the notion of topological entropy using open covers of *X*, another definition of topological entropy was given in [5] which is known as metric entropy. It is proved that these two definitions are equivalent [7].

In the present paper we extend the notion of topological entropy to a finite set of continuous functions on *X* which is called an *Iterated Function System* (IFS) [9,10]. We prove that this extension is invariant under topological conjugate relation for iterated function systems. We show that topological entropy of the inverse of an IFS when it’s elements are homeomorphisms is the same as it’s topological entropy. In section three we present the notion of metric entropy for IFSs, and we prove that it is equal to topological entropy for IFSs on the compact metric spaces. We prove that if F is an IFS, then $h\left(F^{m}\right)=mh\left(F\right)$, where h(.) denotes the topological entropy. If F and G are two IFSs we prove that h(F×G)=h(F)+h(G). We also consider *Average Entropy* as a new approach for topological entropy for an IFS based on the topological entropies of the functions of it. It is also shown that for an IFS, F which all its function satisfies Axiom *A* there exists a neighborhood of F, such that the average entropy of every IFS in this neighborhood is less than or equal to average entropy of F.

## 2. Appearance of Iterated Function Systems in Black Hole Quantum Physics and Bohr-Like Black Hole

Researchers in quantum gravity have the intuitive, common conviction that, in some respects, BHs are the fundamental bricks of quantum gravity in the same way that atoms are the fundamental bricks of quantum mechanics [11]. This similarity suggests that the BH mass should have a discrete spectrum [11]. On the other hand, the analogy generates an immediate and natural question: if the BH is the nucleus of the gravitational atom in quantum gravity, what are the electrons? One of us (Christian Corda) gave an intriguing answer to that question, showing that the BH quasi-normal modes (QNMs) triggered by the emission of Hawking quanta and by the potential absorptions of neighboring particles can be considered as the electrons of that gravitational atom [1,2,3]. Thus, the intuitive picture is more than a picture as QNMs can be really interpreted in terms of BH quantum levels in a BH model somewhat similar to the semi-classical Bohr model of the structure of a hydrogen atom [1,2,3]. This issue has important consequences on the BH information puzzle [12]. In fact, showing BHs in terms of well defined quantum mechanical systems, having an ordered, discrete quantum spectrum, looks consistent with the unitarity of the underlying quantum gravity theory and with the idea that information should come out in BH evaporation [1,2,3]. A fundamental feature of the Bohr-like BH model that we are going to resume is the discreteness of the BH horizon area as the function of the QNMs principal quantum number, which is consistent with various models of quantum gravity where the spacetime is fundamentally discrete [13]. We also stress that, in our knowledge, the first who viewed BHs as similar to gravitational atoms was Bekenstein [11]. In [1,2,3], it has been indeed shown that the semi-classical evaporating Schwarzschild BH is somewhat similar to the historical semi-classical model of the structure of a hydrogen atom introduced by Bohr in 1913. The results in [1,2,3] are founded on the non-thermal spectrum of Parikh and Wilczek [14], which implies the countable character of subsequent emissions of Hawking quanta enabling a natural correspondence between Hawking radiation [15] and the BH quasi-normal modes (QNMs) triggered by the emissions of Hawking quanta and by the potential absorptions of neighbouring particles. In such an approach, those QNMs represent the “electron” which jumps from a level to another one. The absolute values of the QNMs frequencies triggered by emissions (Hawking radiation) and absorption of particles represent, in turn, the energy “shells” of the gravitational hydrogen atom [1,2,3]. Remarkably, the time evolution of BH evaporation is governed by a time-dependent Schrodinger equation and represents an independent approach to solve the BH information puzzle [2,3]. The results in [1,2,3] are also in perfect agreement with previous existing results in the literature, starting from the famous result of Bekenstein on the area quantization [16]. Using Planck units $(G=c=k_{B}=\hbar =\frac{1}{4\pi \epsilon_{0}}=1),$ for large values of the principal quantum number *n* (i.e., for excited BHs), the energy levels of the Schwarzschild BH which is interpreted as gravitational hydrogen atom are given by [1,2,3]

(1) $$E_{n}\equiv \left|\omega_{n}\right|=M-\sqrt{M^{2}-\frac{n}{2}},$$

where *M* is the initial BH mass and $E_{n}$ is interpreted like the total energy emitted when the BH is excited at the level *n* [1,2,3]. During a quantum jump a discrete amount of energy is radiated and, for large values of *n*, the analysis becomes independent of the other quantum numbers, in complete consistence with Bohr’s Correspondence Principle [17], which states that transition frequencies at large quantum numbers should equal classical oscillation frequencies. In Bohr’s model electrons can only gain and lose energy by jumping from one allowed energy shell to another, absorbing or emitting radiation with an energy difference of the levels according to the Planck relation (in standard units) E=hf, where h is the Planck constant and f the transition frequency. In the analysis in [1,2,3] QNMs can only gain and lose energy by jumping from one allowed energy shell to another, absorbing or emitting radiation (emitted radiation is given by Hawking quanta) with an energy difference of the levels according to [1,2,3]

(2) $$\begin{array}{c} \Delta E_{n_{1}\to n_{2}}\equiv E_{n_{2}}-E_{n_{1}}=M_{n_{1}}-M_{n_{2}} \end{array}=\sqrt{M^{2}-\frac{n_{1}}{2}}-\sqrt{M^{2}-\frac{n_{2}}{2}},$$

Equation (2) represents the jump between the two levels $n_{1}$ and $n_{2}>n_{1}$ due to the emission of a particle having frequency $\Delta E_{n_{1}\to n_{2}},$ where $M_{n}$ is the residual mass of the BH excited at the level *n*, that is the original BH mass minus the total energy emitted when the BH is excited at the level *n* [1,2,3]. Thus, $M_{n}=M-E_{n}$ [1,2,3]. Then, the jump between the two levels depends only on the initial BH mass and on the correspondent values of the BH principal quantum number [1,2,3]. In the case of an absorption one gets instead [1,2,3]

(3) $$\begin{array}{c} \Delta E_{n_{2}\to n_{1}}\equiv E_{n_{1}}-E_{n_{2}}=M_{n_{2}}-M_{n_{1}} \end{array}=\sqrt{M^{2}-\frac{n_{2}}{2}}-\sqrt{M^{2}-\frac{n_{1}}{2}}=-\Delta E_{n_{1}\to n_{2}.}$$

The similarity with Bohr’s model is completed if one notes that the interpretation of Equation (3) is of a particle, the electron, quantized on a circle of length [1,2,3]

(4) $$L=4\pi \left(M+\sqrt{M^{2}-\frac{n}{2}}\right),$$

which is the analogous of the electron traveling in circular orbits around the hydrogen nucleus, similar in structure to the solar system, of Bohr model [1,2,3].

The analysis in [1,2,3] permits to show that the famous formula of Bekenstein–Hawking entropy [15,18] is a function of the QNMs principal quantum number, i.e., of the BH quantum level [3]

(5) $${\left(S_{BH}\right)}_{n-1}\equiv \frac{A_{n-1}}{4}=8\pi N_{n-1}M_{n-1}\cdot \Delta E_{n-1\to n}=4\pi \left(M^{2}-\frac{n+1}{2}\right)$$

before the emission and

(6) $${\left(S_{BH}\right)}_{n}\equiv \frac{A_{n}}{4}=8\pi N_{n}M_{n}\cdot \Delta E_{n-1\to n}=4\pi \left(M^{2}-\frac{n}{2}\right)$$

after the emission respectively.

On the other hand, it is a general belief that there is no reason to expect that Bekenstein–Hawking entropy will be the whole answer for a correct quantum gravity theory [3]. For a better understanding of BH entropy we need to go beyond Bekenstein–Hawking entropy and identify the sub-leading corrections [3]. Using the quantum tunneling approach one obtains the sub-leading corrections to the third order approximation [19]. In this approach BH entropy contains four parts: the usual Bekenstein–Hawking entropy, the logarithmic term, the inverse area term and the inverse squared area term [19]

(7) $$S_{total}=S_{BH}-\ln S_{BH}+\frac{3}{2A}+\frac{2}{A^{2}}$$

In this way, the formulas of the total entropy that takes into account the sub-leading corrections to Bekenstein–Hawking entropy become

(8) $$\begin{array}{c} {\left(S_{total}\right)}_{n-1}=4\pi \left(M^{2}-\frac{n-1}{2}\right)-\ln\left[4\pi \left(M^{2}-\frac{n-1}{2}\right)\right] \end{array}+\frac{3}{32\pi \left(M^{2}-\frac{n-1}{2}\right))}+\frac{2}{{\left[16\pi \left(M^{2}-\frac{n-1}{2}\right)\right]}^{2}}$$

before the emission, and

(9) $$\begin{array}{c} {\left(S_{total}\right)}_{n}=4\pi \left(M^{2}-\frac{n}{2}\right)-\ln\left[4\pi \left(M^{2}-\frac{n}{2}\right)\right] \end{array}+\frac{3}{32\pi \left(M^{2}-\frac{n}{2}\right)}+\frac{2}{{\left[16\pi \left(M^{2}-\frac{n}{2}\right)\right]}^{2}}$$

after the emission, respectively. Thus, also the total BH entropy results a function of the BH excited state n. Here we improve the result in [3] where only the second order approximation has been taken into account. We stress that the present results are in perfect agreement with existing results in the literature. In fact, as we consider large n, it is $\Delta E_{n-1\to n}\approx \frac{1}{4M}$, see [3,20] and references within. Thus, if one neglects the difference between the original BH mass and the residual mass $M_{n},$ i.e., $M_{n}≃M$ the Bekenstein–Hawking entropy reads (n≈n−1 and $N_{n}\approx N_{n-1}\equiv N$)

(10) $$S_{BH}=\frac{A}{4}=8\pi NM\cdot \Delta E_{n-1\to n},$$

which is consistent with the standard result, see [3,20] and references within,

(11) $$S_{BH}\to 2\pi N.$$

Again, the consistence with well known and accepted results *cannot* be a coincidence, but it is a confirmation of the correctness of the current analysis instead. Then, the total entropy reads

(12) $$\begin{array}{ll} S_{total} & =8\pi NM\cdot \Delta E_{n-1\to n}-\ln\left[8\pi NM\cdot \Delta E_{n-1\to n}\right]\\ & \;+\frac{3}{64\pi NM\cdot \Delta E_{n-1\to n}}+\frac{2}{{\left(32\pi NM\cdot \Delta E_{n-1\to n}\right)}^{2}} \end{array}$$

which is well approximated by

(13) $$S_{total}≃2\pi N-\ln2\pi N+\frac{3}{16\pi N}+\frac{2}{{\left(8\pi N\right)}^{2}}.$$

Also Equations (12) and (13) improve the results in [3] where only the second order approximation has been taken into account. Now, let us explain the way in which the Bohr-like BH model works following [3]. Let us consider a BH’s original mass M. After an emission from the ground state to a state with large n−1, or, alternatively, after a certain number of emissions (and potential absorptions as the BH can capture neighboring particles), the BH is at an excited level n−1 and its mass is $M_{n-1}\equiv M-E_{n-1}$ where $E_{n-1}$ is the absolute value of the frequency of the QNM associated to the excited level n−1. We recall again that $E_{n-1}$ is interpreted as the total energy emitted at that time [1,2,3]. The BH can further emit an energy to jump to the subsequent level: $\Delta E_{n-1\to n}=E_{n}-E_{n-1}=M_{n-1}-M_{n}$. Now, the BH is at an excited level *n* and the BH mass is

(14) $$\begin{array}{c} M_{n}\equiv M-E_{n-1}-\Delta E_{n-1\to n} \end{array}=M-E_{n-1}+E_{n-1}-E_{n}=M-E_{n}.$$

The BH can, in principle, return to the level n−1 by absorbing an energy $\Delta E_{n\to n-1}=-\Delta E_{n-1\to n}$. In [1,2,3]. it has been also shown that the quantum of area is *the same* for both absorption and emission and it is given by

(15) $$|▵A_{n}|=|▵A_{n-1}|=8\pi ,$$

which is exactly the original result of Bekenstein [16]. Again, we stress that the Bohr-like BH model has important implications for the BH information paradox see [12]. In fact, the results in [1,2,3] show that BH QNMs are really the BH quantum levels in our Bohr-like semi-classical approximation. The time evolution of the Bohr-like BH obeys a *time dependent Schrodinger equation* for the system composed by Hawking radiation and BH QNMs see [2,3]. Such a time evolution enables *pure* quantum states to evolve in *pure* quantum states, while subsequent emissions of Hawking quanta are entangled with BH QNMs [2,3]. On the other hand, consistence between the Bohr-like BH model and a recent approach to solve the BH information paradox [21] has been recently highlighted in [22]. Thus, the general conviction that BHs result in highly excited states representing both the “hydrogen atom” and the “quasi-thermal emission” in an unitary theory of quantum gravity is in perfect agreement with the Bohr-like BH model which seems to approach the final theory of quantum gravity in the same way the Bohr model of hydrogen atom approached the final theory of quantum mechanics.

Appearance of the logarithmic term in Equation (8) implies to the congruence of BH entropy with the metric entropy of a function $f_{n}$ from a state space *X* to itself for a fixed *n* with $1\leq n\leq 2(M^{2}-1)$. The metric entropy can not work when we want to consider different states as a whole. More precisely, the BH entropy depends on *n*. The problem is finding a suitable mathematical model to consider all the *n*-states with $1\leq n\leq 2(M^{2}-1)$ as a system. Our mathematical suggestion for considering this situation is an iterated function system (IFS)

$$F=(X,f_{1},\ldots ,f_{N}),$$

where *X* is a compact metric space. In the classical case we work with autonomous systems, i.e., $f_{1}=f_{2}=\cdots =f_{N}$, but our suggested model is a non-autonomous system.

It is clear that, an iterated function system creates a multifunction with finite range [23].

The orbit of $x_{0}\in X$ corresponding to a sequence ${\left\{i_{n}\right\}}_{n\in N}$ with $i_{n}\in J=\left\{1,\ldots ,N\right\}$ is the sequence ${\left(x_{n}\right)}_{n\in N_{0}}$, where $x_{n}:=f_{i_{n}}\left(x_{n-1}\right)$ and n∈N.

Let α be an open cover for a compact topological space *X*. Then we define:

(16) $$\begin{array}{c} F^{-i}\alpha =\underset{\left[j_{i}\right]\in J^{i}}{⋃}f^{-[j_{i}]}\left(\alpha \right), \end{array}$$

where $\left[j_{i}\right]=\left(j_{1},\ldots ,j_{i}\right)\in J^{i}=\underset{i}{\underset{︸}{J\times \ldots \times J}}$ and $f^{-[j_{i}]}\left(\alpha \right)=f_{j_{1}}^{-1}o\ldots of_{j_{i}}^{-1}\left(\alpha \right)$ for i≥1 and $F^{0}\alpha =\alpha$. It is clear that for each $i\in N_{0}$, $F^{-i}\alpha$ is an open cover for the space *X*.

If N(α) is the number of sets in α with the smallest cardinality (the number of the members) which covers the space *X*, then H(α)=logN(α).

We use of the following lemma.

**Lemma** **1.** *For a given open cover α we have* $H\left(F^{-1}\alpha \right)\leq H\left(\alpha \right)$*. Moreover if* $f_{i}$ *is an onto map for some* 1≤i≤N*, then* $H\left(F^{-1}\alpha \right)=H\left(\alpha \right)$*.*

**Proof.**  Let $\left\{A_{1},\ldots ,A_{n}\right\}$ be a subcover of α for *X*. Then $\left\{F^{-1}\left(A_{1}\right),\ldots ,F^{-1}\left(A_{n}\right)\right\}$ is a subcover of $F^{-1}\alpha$. So $H\left(F^{-1}\alpha \right)\leq H\left(\alpha \right)$. Now, let $f_{i}:X⟶X$ be an onto map, and $\left\{F^{-1}\left(A_{1}\right),\ldots ,F^{-1}\left(A_{n}\right)\right\}$ be a subcover of $F^{-1}\alpha$. Then $\left\{A_{1},\ldots ,A_{n}\right\}$ is a subcover of α. Hence $H\left(\alpha \right)\leq H\left(F^{-1}\alpha \right)$. ☐

For two open covers $\alpha =\{A_{1},\ldots ,A_{n}\}$ and $\beta =\{B_{1},\ldots ,B_{m}\}$, we define

$$\begin{array}{c} F^{-1}\left(\alpha \vee \beta \right)=\left\{f_{k}^{-1}\left(A_{i}\cap B_{j}\right):1\leq k\leq N,1\leq i\leq n,1\leq j\leq m\right\}, \end{array}$$

and

$$\begin{array}{c} F^{-1}\left(\alpha \right)\vee F^{-1}\left(\beta \right)=\left\{f_{k}^{-1}\left(A_{i}\right)\cap f_{L}^{-1}\left(B_{j}\right):1\leq L,k\leq N,1\leq i\leq n,1\leq j\leq m\right\}. \end{array}$$

If α and β are two open covers for the space *X*, then the open cover α is called a refinement of β if each member of α is a subset of a member of β. In this case we write β≺α.

Let $f_{k}^{-1}\left(A_{i}\cap B_{j}\right)$ be a member of $F^{-1}\left(\alpha \vee \beta \right)$. Then $f_{k}^{-1}\left(A_{i}\cap B_{j}\right)\subseteq f_{k}^{-1}\left(A_{i}\right)\cap f_{k}^{-1}\left(B_{j}\right)$. Thus $F^{-1}\left(\alpha \right)\vee F^{-1}\left(\beta \right)≺F^{-1}\left(\alpha \vee \beta \right)$ for each two covers α,β. So we have $N\left(F^{-1}\left(\alpha \right)\vee F^{-1}\left(\beta \right)\right)\leq N\left(F^{-1}\left(\alpha \vee \beta \right)\right).$ It is not necessary that $F^{-1}\left(\alpha \right)\vee F^{-1}\left(\beta \right)=F^{-1}\left(\alpha \vee \beta \right),$ (see Example 1). Similarly we have $F^{-i}\left(\alpha \right)\vee F^{-i}\left(\beta \right)≺F^{-i}\left(\alpha \vee \beta \right)$, for each i≥0, and

$$\begin{array}{c} \vee_{j=0}^{n-1}F^{-k}\left(\alpha_{j}\right)≺F^{-k}\left(\vee_{j=0}^{n-1}\left(\alpha_{j}\right)\right). \end{array}$$

Thus

(17) $$\begin{array}{c} H\left(\vee_{j=0}^{n-1}F^{-k}\left(\alpha_{j}\right)\right)\leq H\left(F^{-k}\left(\vee_{j=0}^{n-1}\left(\alpha_{j}\right)\right)\right), \end{array}$$

for every finite covers $\alpha_{0},\;\alpha_{2},\;\ldots ,\;\alpha_{n-1}$.

The following example shows that the converse of the Inequality (17) is not always true.

**Example** **1.** *Consider the IFS* F=(X,f,g) *where* X=[0,1] *and* f,g:X⟶X *are defined by* f(x)=1−x *and* $g\left(x\right)=x^{2}$*. If* $\alpha =\{\left[0,\frac{1}{2}\right),\left[\frac{1}{2},1\right]\}$ *and* $\beta =\{\left[0,\frac{1}{3}\right),\left[\frac{1}{3},1\right]\}$ *on X, then we have*

$$\begin{array}{c} F^{-1}\left(\alpha \vee \beta \right)=\left\{\left(\frac{2}{3},1\right],\left(\frac{1}{2},\frac{2}{3}\right],\left[\frac{1}{2},\frac{2}{3}\right],\left[0,\frac{1}{3}\right),\left[\frac{1}{\sqrt{3}},\frac{1}{\sqrt{2}}\right),\left[\frac{1}{\sqrt{3}},\frac{1}{\sqrt{2}}\right]\right\} \end{array}$$

*but*

$$\begin{array}{c} F^{-1}\left(\alpha \right)\vee F^{-1}\left(\beta \right)=\{\left(\frac{2}{3},1\right],\left(\frac{1}{2},\frac{2}{3}\right],\left(\frac{1}{2},1\right],\left[0,\frac{1}{2}\right],\\ \left(\frac{2}{3},\frac{1}{\sqrt{2}}\right),\left[0,\frac{2}{3}\right],\left[0,\frac{1}{\sqrt{3}}\right),\left[\frac{1}{\sqrt{3}},\frac{1}{\sqrt{2}}\right),\left[\frac{1}{\sqrt{2}},1\right]\}. \end{array}$$

**Lemma** **2.** $\lim_{n\to \infty }\frac{1}{n}H\left(\vee_{i=0}^{n-1}F^{-i}\alpha \right)$ *exists.*

**Proof.**  Consider the sequence ${\left(a_{n}\right)}_{n\in N}$ which $a_{n}=H\left(\vee_{i=0}^{n-1}F^{-i}\alpha \right)$ for all n∈N. Then for each k,n∈N we have

$$\begin{array}{cc} a_{n+k} & =H(\vee_{i=0}^{n+k-1}F^{-i}\alpha )\\ & \leq H\left(\vee_{i=0}^{n-1}F^{-i}\alpha \right)+H\left(\vee_{i=n}^{n+k-1}F^{-i}\alpha \right)\\ & =H\left(\vee_{i=0}^{n-1}F^{-i}\alpha \right)+H\left(\vee_{i=0}^{k-1}F^{-i-n}\alpha \right)\\ & \leq H\left(\vee_{i=0}^{n-1}F^{-i}\alpha \right)+H\left(F^{-n}\left(\vee_{i=0}^{k-1}F^{-i}\alpha \right)\right)\;\text{Inequality (17)}\\ & \leq H\left(\vee_{i=0}^{n-1}F^{-i}\alpha \right)+H\left(\vee_{i=0}^{k-1}F^{-i}\alpha \right)\;\text{Lemma 1}\;\\ & =a_{n}+a_{k}. \end{array}$$

So $a_{n+k}\leq a_{n}+a_{k}.$ Thus $a_{n+k}$ is a subadditive sequence [7]. Hence we have $\lim_{n\to \infty }\frac{1}{n}H\left(\vee_{i=0}^{n-1}F^{-i}\alpha \right)=\lim_{n\to \infty }\frac{a_{n}}{n}$. ☐

Now we define the topological entropy of F, based on the open covers of *X*.

**Definition** **1.** *We define the topological entropy of* F *relative to α by:*

$$\begin{array}{c} h_{\tau }\left(F,\alpha \right)=\lim_{n\to \infty }\frac{1}{n}H\left(\vee_{i=0}^{n-1}F^{-i}\alpha \right), \end{array}$$

*and the topological entropy* F *by*

$$\begin{array}{c} h_{\tau }\left(F\right)=\underset{\alpha }{\sup}h_{\tau }\left(F,\alpha \right). \end{array}$$

This is well known that topological entropy is an invariant of topological conjugacy. Now we define topological conjugacy for iterated function systems and in Theorem 1 we prove the same result for iterated function systems.

Let $\left(X,\tau_{1}\right)$ and $\left(Y,\tau_{2}\right)$ be two compact topological spaces and J={1,…,N} be a finite set. If $F=(X,f_{1},\ldots ,f_{N})$ and $G=(Y,g_{1},\ldots ,g_{N})$ are two IFSs, then we say that F is topologically conjugate to G if there is a homeomorphism ϕ:X⟶Y such that $\phi of_{i}=g_{i}o\phi$, for all i∈J.

**Remark** **1.** *Let α be an open cover for X and let* ϕ:X⟶X *be an onto continuous map. Then* $H\left(\phi^{-1}\alpha \right)=H\left(\alpha \right)$ *(Remark 5, Chapter 5, [7]).*

**Theorem** **1.** *With the above assumptions, if* F *and* G *are topologically conjugate then* $h_{\tau_{1}}\left(F\right)=h_{\tau_{2}}\left(G\right).$

**Proof.**  Since $\phi of_{i}=g_{i}o\phi$, for all 1≤i≤N, then by Remark 1 we have

$$\begin{array}{cc} h_{\tau_{2}}\left(G,\alpha \right) & =\lim_{n\to \infty }\frac{1}{n}H\left(\vee_{i=0}^{n-1}G^{-i}\alpha \right)\\ & =\lim_{n\to \infty }\frac{1}{n}H\left(\phi^{-1}\left(\vee_{i=0}^{n-1}G^{-i}\alpha \right)\right)\;\mathrm{by}\;\mathrm{Remark}\;1\\ & =\lim_{n\to \infty }\frac{1}{n}H\left(\vee_{i=0}^{n-1}\phi^{-1}\left(G^{-i}\alpha \right)\right)\\ & =\lim_{n\to \infty }\frac{1}{n}H\left(\vee_{i=0}^{n-1}F^{-i}\left(\phi^{-1}\alpha \right)\right)\;\mathrm{Because}\;\phi of_{i}=g_{i}o\phi ,\\ & =h_{\tau_{1}}\left(F,\phi^{-1}\left(\alpha \right)\right). \end{array}$$

Hence $h_{\tau_{1}}\left(F\right)\geq h_{\tau_{2}}\left(G\right).$ Similarly, by replacing ϕ with $\phi^{-1}$ we have $h_{\tau_{2}}\left(G\right)\geq h_{\tau_{1}}\left(F\right)$. So $h_{\tau_{1}}\left(F\right)=h_{\tau_{2}}\left(G\right).$ ☐

**Theorem** **2.** *Let* $F=(X,f_{1},\ldots ,f_{N})$ *be an* IFS*, and let* $f_{1},\ldots ,f_{N}:X⟶X$ *be homeomorphisms. Then* $h_{\tau }\left(F\right)=h_{\tau }\left(F^{-1}\right)$*, where the* $IFS\;F^{-1}$ *is defined by:*

$$\begin{array}{c} F^{-1}:=\left(X,f_{1}^{-1},\ldots ,f_{N}^{-1}\right). \end{array}$$

**Proof.**  $$\begin{array}{cc} h_{\tau }\left(F^{-1},\alpha \right) & =\lim_{n\to \infty }\frac{1}{n}H\left(\vee_{i=0}^{n-1}F^{i}\alpha \right)\;\\ & \leq \lim_{n\to \infty }\frac{1}{n}H\left(F^{n-1}\left(\vee_{i=0}^{n-1}F^{i}\alpha \right)\right)\;\mathrm{by}\;\mathrm{Lemma}\;1\\ & =\lim_{n\to \infty }\frac{1}{n}H\left(\vee_{i=0}^{n-1}F^{-i}\alpha \right)\\ & =h_{\tau }\left(F,\alpha \right). \end{array}$$

So $h_{\tau }\left(F^{-1}\right)\leq h_{\tau }\left(F\right)$. Similarly we have $h_{\tau }\left(F\right)\leq h_{\tau }\left(F^{-1}\right)$. Thus $h_{\tau }\left(F\right)=h_{\tau }\left(F^{-1}\right)$. ☐

## 3. Metric Entropy

Let $F=(X,f_{1},\ldots ,f_{N})$ be an IFS with continuous maps $\left\{f_{i}\right\}$. For a given n>1, we define a metric $d_{n}$ on *X* by:

(18) $$\begin{array}{c} d_{n}\left(x,y\right)=\max_{\left[j_{i}\right]\in J^{i}}\left\{d\left(x,y\right),d\left(f^{\left[j_{i}\right]}\left(x\right),f^{\left[j_{i}\right]}\left(y\right)\right)\right\}, \end{array}$$

where $\left[j_{i}\right]=\left(j_{1},\ldots ,j_{i}\right)\in J^{i}$, 1≤i≤n−1 and $f^{\left[j_{i}\right]}\left(x\right)=f_{j_{i}}o\ldots .of_{j_{1}}\left(x\right)$.

A neighborhood of *x* with the radius ϵ with respect to $d_{n}$ is:

$$\begin{array}{c} \underset{0\leq i\leq n-1}{⋂}f^{-[j_{i}]}\left(N\left(f^{\left[j_{i}\right]}\left(x\right),\epsilon \right)\right), \end{array}$$

where $\left(j_{1},\ldots ,j_{i}\right)\in J^{i}$, $f^{\left[j_{0}\right]}=f^{-[j_{0}]}=I_{X}$ and N(x,ϵ) is a neighborhood of *x* with the radius ϵ with respect to *d*.

Let *K* be a compact subset of *X*. A subset *E* of *K* is called (n,ϵ)-separated if for each x,y∈E we have x=y or $d_{n}\left(x,y\right)>\epsilon$. s(n,ϵ,K,F) denotes the largest cardinality of (n,ϵ)-separated sets of *K*.

A subset *W* of *X* is called (n,ϵ)-spanning set for a compact subset *K*, if for every x∈K there is a y∈W with $d_{n}\left(x,y\right)\leq \epsilon$. r(n,ϵ,K,F) denotes the smallest cardinality of (n,ϵ)-spanning sets of *K*.

Now we present the notion of *metric entropy* for IFS.

**Definition** **2.** *The metric entropy of an* IFS $F=(X,f_{1},\ldots ,f_{N})$ *is:*

$$\begin{array}{cc} h_{d}\left(F\right) & =\underset{K\mathrm{is}\;\mathrm{compact}}{\sup}\lim_{\epsilon \to 0}\underset{n\to \infty }{lim sup}\frac{1}{n}\log\left(s\left(n,\epsilon ,K,F\right)\right)\\ & =\underset{K\mathrm{is}\;\mathrm{compact}}{\sup}\lim_{\epsilon \to 0}\underset{n\to \infty }{lim sup}\frac{1}{n}\log\left(r\left(n,\epsilon ,K,F\right)\right) \end{array}$$

Next theorem shows that the metric entropy and the topological entropy of an IFS are equal.

**Theorem** **3.** *If* $F=(X,f_{1},\ldots ,f_{N})$ *is an* IFS *on the compact metric X, then* $h_{\tau }\left(F\right)=h_{d}\left(F\right)$*.*

**Proof.**  Suppose that α is a finite open cover for *X* and diam(α)=sup{d(A):A∈α}≤ϵ, where d(A)=sup{d(x,y):x,y∈A}. Let *E* be an (n,ϵ)-separated set with the cardinality s(n,ϵ,F) and let x,y be two distinct members of *E*. Since $d_{n}\left(x,y\right)>\epsilon$ then x,y can not lay in the one member of $\vee_{i=0}^{n-1}F^{-i}\left(\alpha \right)$, so $s\left(n,\epsilon ,F\right)\leq N(\vee_{i=0}^{n-1}F^{-i}\left(\alpha \right))$. Hence $h_{d}\left(F\right)\leq h_{\tau }\left(F\right)$.

Now we prove $h_{d}\left(F\right)\geq h_{\tau }\left(F\right)$. Let α be an open cover of *X* with the Lebesgue number δ. For an $\left(n,\frac{\delta }{2}\right)$-spanning set *W* with the cardinality $r(n,\frac{\delta }{2},F)$ we have

$$\begin{array}{c} X=\underset{x\in W}{⋃}\underset{0\leq i\leq n-1}{⋂}f^{-[j_{i}]}(\bar{N}\left(f^{\left[j_{i}\right]}\left(x\right),\frac{\delta }{2}\right), \end{array}$$

where $\left(j_{1},\ldots ,j_{i}\right)\in J^{i}$, $f^{\left[j_{0}\right]}=f^{-[j_{0}]}=I_{X}$. Since for $\left(j_{1},\ldots ,j_{i}\right)\in J^{i}$ there exists a member A∈α such that

$$\begin{array}{c} \bar{N}\left(f^{\left[j_{i}\right]}\left(x\right),\frac{\delta }{2}\right)\subseteq A, \end{array}$$

then

$$\begin{array}{c} \underset{0\leq i\leq n-1}{⋂}f^{-[j_{i}]}\left(\bar{N}\left(f^{\left[j_{i}\right]}\left(x\right),\frac{\delta }{2}\right)\right)\subseteq A\cap f_{j_{1}}^{-1}of_{j_{0}}^{-1}\left(A\right)\cap \ldots \cap f_{j_{i}}^{-1}o\ldots of_{j_{0}}^{-1}\left(A\right). \end{array}$$

Hence $N\left(\vee_{i=0}^{n-1}F^{-i}\alpha \right)\leq r\left(n,\frac{\delta }{2},F\right).$ This implies that $h_{\tau }\left(F\right)\leq h_{d}\left(F\right)$. ☐

We write $h_{\tau }\left(F\right)=h_{d}\left(F\right)=h\left(F\right)$.

It is well known that for every continuous map f:X→X, the power rule for its entropy holds, i.e., $h\left(f^{m}\right)=mh\left(f\right)$ for any positive integer *m*. By Theorem 4 we prove a similar result for IFS.

**Definition** **3.** *If* $F=(X,f_{1},\ldots ,f_{N})$ *is an* IFS *[24]. Then we define the* IFS $F^{m}$ *by:*

$$\begin{array}{c} F^{m}:=\left(X,f_{I_{1}},\ldots ,f_{I_{N^{m}}}\right), \end{array}$$

*where* $f_{I_{i}}=f_{i_{m}}o\ldots of_{i_{1}}$ *for all* $I_{i}=\left(i_{1},\ldots ,i_{m}\right)\in J^{m}$ *and* $1\leq i\leq N^{m}$*.*

**Lemma** **3.** *Every* (mn,ϵ)*-spanning set of an* IFS F*, is a* (n,ϵ)*-spanning set for the* IFS $F^{m}$*.*

**Proof.**  Let *W* be an (mn,ϵ)-spanning set for an IFS F. Then for every x≠y, x,y∈W we have

$$\begin{array}{c} \max_{\left[j_{i}\right]\in J^{i}}\left\{d\left(x,y\right),d\left(f^{\left[j_{i}\right]}\left(x\right),f^{\left[j_{i}\right]}\left(y\right)\right)\right\}\leq \epsilon , \end{array}$$

where $\left[j_{i}\right]=\left(j_{1},\ldots ,j_{i}\right)\in J^{i}$, 1≤i≤mn−1 and $f^{\left[j_{i}\right]}\left(x\right)=f_{j_{i}}o\ldots .of_{j_{1}}\left(x\right)$.

Hence

$$\begin{array}{c} \max_{\left[I_{i}\right]}\left\{d\left(x,y\right),d\left(f^{\left[I_{i}\right]}\left(x\right),f^{\left[I_{i}\right]}\left(y\right)\right)\right\}\leq \epsilon , \end{array}$$

where $I_{k}\in J^{m}$, 1≤k≤n−1, $\left[I_{i}\right]=\left(I_{1},\ldots ,I_{i}\right)$ and $f^{\left[I_{i}\right]}\left(x\right)=f_{I_{i}}o\ldots .of_{I_{1}}\left(x\right)$. So *W* is an (n,ϵ)-spanning set for the IFS $F^{m}$. ☐

**Theorem** **4.** *Suppose that* $F=(X,f_{1},\ldots ,f_{N})$ *is an* IFS*, where* $f_{1},\ldots ,f_{N}:X⟶X$ *are continuous maps, and* m∈N*, then* $h\left(F^{m}\right)=mh\left(F\right)$*.*

**Proof.**  By Lemma 3 each (n,ϵ)-spanning set of an IFS $F^{m}$ is an (mn,ϵ)-spanning set of F. So $r\left(n,\epsilon ,K,F^{m}\right)\leq r\left(mn,\epsilon ,K,F\right)$. Thus $h\left(F^{m}\right)\leq mh\left(F\right).$

Now, we prove the other inequality. Since each $f_{i}$ is continuous and *X* is compact, then for ϵ>0 there is a δ>0 such that

$$\begin{array}{c} d\left(x,y\right)<\delta ⟹d(f^{\left[j_{i}\right]}\left(x\right),f^{\left[j_{i}\right]}\left(y\right))<\epsilon , \end{array}$$

for all 0≤i≤m−1. If *E* is a (n,δ)-spanning set for *K* with respect to the IFS $F^{m}$, then for each x∈K there is y∈E such that

$$\begin{array}{c} d(x,y)<\delta \\ d(f^{\left[I_{1}\right]}\left(x\right),f^{\left[I_{1}\right]}\left(y\right))<\delta \\ d(f^{\left[I_{2}\right]}\left(f^{\left[I_{1}\right]}\left(x\right)\right),f^{\left[I_{2}\right]}\left(f^{\left[I_{1}\right]}\left(y\right)\right))<\delta \\ .\\ .\\ .\\ d(f^{\left[I_{n-1}\right]}o\ldots o(f^{\left[I_{1}\right]}\left(x\right),f^{\left[I_{n-1}\right]}o\ldots o\left(f^{\left[I_{1}\right]}\left(y\right)\right)<\delta , \end{array}$$

where $I_{i}\in J^{m}$ and 1≤i≤n−1. So

$$\begin{array}{c} d\left(x,y\right)<\epsilon \;\mathrm{and}\;d(f^{\left[j_{i}\right]}\left(x\right),f^{\left[j_{i}\right]}\left(y\right))<\epsilon , \end{array}$$

for every $\left[j_{i}\right]=\left(j_{1},\ldots ,j_{i}\right)\in J^{i}$, 1≤i≤nm−1. Hence every (n,δ)-spanning set of *K* with respect to the IFS $F^{m}$ is a (nm,ϵ)-spanning set of *K* with respect to the IFS F. Therefore $r\left(n,\delta ,K,F^{m}\right)\geq r\left(mn,\epsilon ,K,F\right)$, this implies that $m{lim sup}_{n\to \infty }\frac{r(mn,\epsilon ,K,F)}{mn}\leq lim sup\frac{r(n,\delta ,K,F^{m})}{n}$. Thus $mh\left(F\right)\leq h\left(F^{m}\right).$ ☐

This is well known that if f,g:X→X are two continuous functions then h(f×g)=h(f)+h(g). Now we consider the product of two IFS and prove the similar property.

**Definition** **4.** *Suppose that* $\left(X,d_{1}\right)$ *and* $\left(Y,d_{2}\right)$ *are two compact metric spaces, and* $F=(X,f_{1},\ldots ,f_{N})$*,* $G=(Y,g_{1},\ldots ,g_{M})$ *are two* IFSs*. Then the product of* F,G *is defined by:*

$$\begin{array}{c} F\times G=(X\times Y,f_{j}\times g_{i}), \end{array}$$

*where* j∈J={1,…,N} *and* i∈L={1,…,M}*. Additionally,* (X×Y,d) *is a compact metric space, where* $d\left(\left(x_{1},x_{2}\right),\left(y_{1},y_{2}\right)\right):=\max\left\{d_{1}\left(x_{1},y_{1}\right),d_{2}\left(x_{2},y_{2}\right)\right\}.$

**Theorem** **5.** *Let* $\left(X,d_{1}\right)$ *and* $\left(Y,d_{2}\right)$ *be two compact metric spaces, and* $F=(X,f_{1},\ldots ,f_{N})$*,* $G=(Y,g_{1},\ldots ,g_{M})$ *are two* IFS*. Then*

$$\begin{array}{c} h(F\times G)=h(F)+h(G). \end{array}$$

**Proof.**  Consider $W_{1}$ and $W_{2}$ as two (n,ϵ)-spanning sets for F and G respectively. For each $x_{1}\in X,\;x_{2}\in Y$ there are $y_{1}\in W_{1},\;y_{2}\in W_{2}$ such that

$$\begin{array}{c} d_{1}\left(x_{1},y_{1}\right)<\epsilon ,\;d_{1}\left(f^{\left[j_{i}\right]}\left(x_{1}\right),f^{\left[j_{i}\right]}\left(y_{1}\right)\right)\leq \epsilon , \end{array}$$

and

$$\begin{array}{c} d_{2}\left(x_{2},y_{2}\right)<\epsilon ,\;d_{2}\left(f^{\left[l_{i}\right]}\left(x_{2}\right),f^{\left[l_{i}\right]}\left(y_{2}\right)\right)\leq \epsilon , \end{array}$$

where $\left[j_{i}\right]=\left(j_{1},\ldots ,j_{i}\right)\in J^{i}$, 1≤i≤n−1 and $\left[l_{i}\right]=\left(l_{1},\ldots ,l_{i}\right)\in L^{i}$, 1≤i≤n−1 and L={1,…,M}. If we take $F\times G=(X\times Y,h_{1},\ldots ,h_{NM})$ with $h_{i}=f_{s_{i}}\times g_{t_{i}}$ then

$$\begin{array}{c} \;d(h_{r_{i}}o\ldots oh_{r_{0}}\left(x_{1},x_{2}\right),h_{r_{i}}o\ldots oh_{r_{0}}\left(y_{1},y_{2}\right))\\ =\max\{d_{1}\left(f^{\left[j_{i}\right]}\left(x_{1}\right),f^{\left[j_{i}\right]}\left(y_{1}\right)\right),d_{2}\left(g^{\left[l_{i}\right]}\left(x_{2}\right),g^{\left[l_{i}\right]}\left(y_{2}\right)\right)\}\leq \epsilon , \end{array}$$

where 0≤i≤n−1, $\left(j_{1},\ldots ,j_{i}\right)\in J^{i}$, $\left(l_{1},\ldots ,l_{i}\right)\in L^{i}$, $f^{\left[j_{0}\right]}=I_{X}$ and $g^{\left[l_{0}\right]}=I_{Y}$. Hence $W_{1}\times W_{2}$ is an (n,ϵ)-spanning set for the IFSF×G and consequently r(n,ϵ,X×Y,F×G)≤r(n,ϵ,X,F)×r(n,ϵ,Y,G). Thus h(F×G)≤h(F)+h(G). If $E_{1}$ and $E_{2}$ are (n,ϵ)-separated subsets of *X* and *Y* respectively, then $E_{1}\times E_{2}$ is an (n,ϵ)-separated subset of X×Y. Thus s(n,ϵ,X×Y,F×G)≥s(n,ϵ,X,F)×s(n,ϵ,Y,G), and we have h(F×G)≥h(F)+h(G). ☐

In the following example we compute topological entropy for an IFS.

**Example** **2.** *Suppose that* F=(R,2x,3x)*. By using of Formula (18) we have*

$$\begin{array}{cc} d_{n}\left(x,y\right) & =\max_{\left[j_{i}\right]\in J^{i}}\left\{d\left(x,y\right),d\left(f^{\left[j_{i}\right]}\left(x\right),f^{\left[j_{i}\right]}\left(y\right)\right)\right\}\\ & =\max_{0\leq i\leq n-1}\left\{d\left(x,y\right),d\left(2^{i}\times 3^{n-1-i}\times x,2^{i}\times 3^{n-1-i}\times y\right)\right\}\\ & =\max_{0\leq i\leq n-1}\{|x-y|,|2^{i}\times 3^{n-1-i}\times x-2^{i}\times 3^{n-1-i}\times y\left|\right\}\\ & =\max_{0\leq i\leq n-1}\left\{d\left(x,y\right),\right|2^{i}\times 3^{n-1-i}\times x-2^{i}\times 3^{n-1-i}\times y\left|\right\}\\ & =3^{n-1}\left|x-y\right|. \end{array}$$

*where* $j_{i}=\left(j_{1},\ldots ,j_{i}\right)\in J^{i}$*,* 1≤i≤n−1 *and* $f^{\left[j_{i}\right]}\left(x\right)=f_{j_{i}}o\ldots .of_{j_{1}}\left(x\right)$*. Now suppose K is a compact subset of* R *with* sup{d(x,y):x,y∈K}=r *and E is an* (n,ϵ)*-separated subset of K. Since* $x,y\in E⟺|x-y|>\frac{\epsilon }{3^{n-1}}$*, then* $s\left(n,\epsilon ,K,F\right)\leq \frac{r\times 3^{n-1}}{\epsilon }$*, and* $h\left(F\right)=\lim_{\epsilon \to 0}{lim sup}_{n\to \infty }\frac{1}{n}\log\left(\frac{r\times 3^{n-1}}{\epsilon }\right)=\log3$*, hence* h(F)=log3*.*

Similar calculations imply that if F=(R,ax,bx), then h(F)=logb, where b≥a>1.

## 4. Average Entropy

In this section we present another method to define the entropy of an IFS.

Let *X* be a topological space, and let $F=(X,f_{1},\ldots ,f_{N})$ be an IFS on *X*, where $f_{i}:X⟶X$, i∈J={1,…,N} are distinct continuous maps. A typical element of $J^{N}$ can be denoted by $\sigma =\{\lambda_{1},\lambda_{2},\ldots \}$ and we use the notation $F_{\sigma_{n}}=f_{\lambda_{n}}of_{\lambda_{n-1}}o\ldots of_{\lambda_{1}},$ for n∈N. $F_{\sigma }=\left\{F_{\sigma_{n}}:n\geq 1\right\}$ and we denote the set of $F_{\sigma }=\left\{F_{\sigma_{n}}:n\geq 1\right\}$ by A(F).

**Definition** **5.** *For an* IFS*,* $F=(X,f_{1},\ldots ,f_{N})$*, and* $\sigma =\{\lambda_{1},\lambda_{2},\ldots \}$ *we define the topological entropy of* $F_{\sigma }$ *by:*

(19) $$\begin{array}{c} h\left(F_{\sigma }\right)=\sum_{k=1}^{N}b_{k}h\left(f_{k}\right), \end{array}$$

*where* $b_{k}={lim sup}_{n\to \infty }\frac{\sum_{i=1}^{n}\delta \left(\lambda_{i}=k\right)}{n}$*,* $\delta \left(\lambda_{i}=k\right)=\left\{\begin{array}{cc} 1 & if\lambda_{i}=k\\ \;0 & if\;\lambda_{i}\neq k \end{array},\right.$ *and* $h(f_{k})$ *is the topological entropy of the* $f_{k}$*.*

**Lemma** **4.** *Let* ${\left\{a_{i}\right\}}_{i=1}^{\infty }$ *and* ${\left\{b_{k}\right\}}_{k=1}^{N}$ *be two sequences of positive numbers. Then*

$$\begin{array}{c} \sum_{k=1}^{N}\left(\underset{n\to \infty }{lim sup}\frac{\sum_{i=1}^{n}a_{i}}{n}\right)b_{k}\geq \underset{n\to \infty }{lim sup}\sum_{k=1}^{N}\frac{\sum_{i=1}^{n}a_{i}b_{k}}{n} \end{array}$$

**Proof.**  For fixed $b_{k}$

$$\begin{array}{cc} \frac{\sum_{i=1}^{n}a_{i}b_{k}}{n} & =\left(\frac{\sum_{i=1}^{n}a_{i}}{n}\right)b_{k}\\ & \leq \left(\underset{n\to \infty }{lim sup}\frac{\sum_{i=1}^{n}a_{i}}{n}\right)b_{k}. \end{array}$$

Thus

$$\begin{array}{c} \sum_{k=1}^{N}\left(\frac{\sum_{i=1}^{n}a_{i}}{n}\right)b_{k}\leq \sum_{k=1}^{N}\left(\underset{n\to \infty }{lim sup}\frac{\sum_{i=1}^{n}a_{i}}{n}\right)b_{k}. \end{array}$$

So

$$\begin{array}{c} \underset{n\to \infty }{lim sup}\sum_{k=1}^{N}\left(\frac{\sum_{i=1}^{n}a_{i}}{n}\right)b_{k}\leq \sum_{k=1}^{N}\left(\underset{n\to \infty }{lim sup}\frac{\sum_{i=1}^{n}a_{i}}{n}\right)b_{k}. \end{array}$$

☐

**Theorem** **6.** *Suppose that* $F=(X,f_{1},\ldots ,f_{N})$ *is an* IFS *then for every* $\sigma =\left\{\lambda_{1},\lambda_{2},\ldots \right\}\in J^{N}$

$$\begin{array}{c} \min\left\{h\left(f_{k}\right):k\in J\right\}\leq \underset{n\to \infty }{lim sup}\sum_{i=1}^{n}\frac{h(f_{\lambda_{i}})}{n}\leq h\left(F_{\sigma }\right)\leq \sum_{k=1}^{N}h\left(f_{k}\right). \end{array}$$

**Proof.**  $$\begin{array}{cc} h(F_{\sigma }) & =\sum_{k=1}^{N}\left(\underset{n\to \infty }{lim sup}\frac{\sum_{i=1}^{n}\delta \left(f_{\lambda_{i}}=f_{k}\right)}{n}\right)h\left(f_{k}\right)\\ & \geq \underset{n\to \infty }{lim sup}\sum_{k=1}^{N}\frac{\sum_{i=1}^{n}\delta \left(f_{\lambda_{i}}=f_{k}\right)h\left(f_{k}\right)}{n}\;\mathrm{by}\;\mathrm{Lemma}\;4\\ & =\underset{n\to \infty }{lim sup}\sum_{i=1}^{n}\frac{\sum_{k=1}^{N}\delta \left(f_{\lambda_{i}}=f_{k}\right)h\left(f_{k}\right)}{n}\\ & =\underset{n\to \infty }{lim sup}\sum_{i=1}^{n}\frac{h(f_{\lambda_{i}})}{n}. \end{array}$$

For every m∈N we have

$$\begin{array}{cc} \underset{n\to \infty }{lim sup}\sum_{i=1}^{n}\frac{h(f_{\lambda_{i}})}{n} & \geq \sum_{i=1}^{m}\frac{h(f_{\lambda_{i}})}{m}\\ & \geq \min\{h\left(f_{k}\right):k\in J\}. \end{array}$$

Since for all k∈N we have $b_{k}\leq 1$, then

(20) $$\begin{array}{c} h\left(F_{\sigma }\right)=\sum_{k=1}^{N}b_{k}h\left(f_{k}\right)\leq \sum_{k=1}^{N}h\left(f_{k}\right). \end{array}$$

☐

**Example** **3.** *Let* $F=(R,f_{1}\left(x\right)=2x,f_{2}\left(x\right)=3x)$ *and let*

$$\begin{array}{c} \sigma =\{1,\;2,\;1,\;1,\;2,\;2,\;1,\;1,\;1,\;1,\;2,\;2,\;2,\;2,\;\ldots .\}. \end{array}$$

*Put* $A_{n}=\sum_{i=1}^{n}\delta \left(f_{\lambda_{i}}=2x\right)$ *and* $B_{n}=\sum_{i=1}^{n}\delta \left(f_{\lambda_{i}}=3x\right)$*,* n≥1*.*

*Then*

$$\begin{array}{c} A_{3\times 2^{k}-2+i}=2^{k+1}-1,\;B_{3\times 2^{k}-2+i}=2^{k}-1+i,\;0\leq i\leq 2^{k}\\ A_{3\times 2^{k}-2+2^{k}+i}=2^{k+1}-1+i,\;B_{3\times 2^{k}-2+2^{k}+i}=2^{k+1}-i,\;0\leq i\leq 2^{k+1}-1. \end{array}$$

*So*

$$\begin{array}{c} \underset{n\to \infty }{lim sup}\frac{A_{n}}{n}=\frac{2}{3},\;\underset{n\to \infty }{lim sup}\frac{B_{n}}{n}=\frac{1}{2}. \end{array}$$

*Thus*

$$\begin{array}{c} h\left(F_{\sigma }\right)=\frac{2}{3}\log2+\frac{1}{2}\log3. \end{array}$$

The following theorem is the main result of this paper. It gives a new type of topological entropy for an IFS, $F=(X,f_{1},\ldots ,f_{N})$ based on the usual topological entropy of $f_{1},\ldots ,f_{N}$.

**Theorem** **7.** *Let* $F=(X,f_{1},\ldots ,f_{N})$ *be an* IFS*, then*

$$\begin{array}{c} \sup\left\{h\left(F_{\sigma }\right):\sigma \in J^{N}\right\}=\sum_{k=1}^{N}h\left(f_{k}\right). \end{array}$$

**Proof.**  Let $F=(X,f_{1},\ldots ,f_{N})$ be an IFS with distinct continuous maps $f_{1},\ldots ,f_{N}$. Define the maps ${\left(f_{j}^{\prime }\right)}_{j\in N}$ by:

$$\begin{array}{c} f_{j}^{\prime }=f_{i},\;j\overset{N}{≅}i. \end{array}$$

We define ${\left(g_{k}\right)}_{j\in N}$ by:

$$\begin{array}{c} g_{1}=f_{1}^{\prime },\\ g_{2}=f_{2}^{\prime },\\ \underset{2^{2}}{\underset{︸}{g_{3}=g_{4}=\ldots =g_{6}}}=f_{3}^{\prime },\\ \underset{6^{2}}{\underset{︸}{g_{7}=\ldots =g_{7+6^{2}-1}}}=f_{4}^{\prime },\\ \underset{{\left(7+6^{2}-1\right)}^{2}}{\underset{︸}{g_{7+6^{2}}=\ldots =g_{7+6^{2}+{\left(7+6^{2}-1\right)}^{2}-1}}}=f_{5}^{\prime }\\ .\\ .\\ . \end{array}$$

This means that for each n≥2, $g_{n_{1}}=g_{n_{1}+1}=\ldots .=g_{n_{2}}=f_{n}^{\prime }$. So

$$\underset{{\left(n_{2}\right)}^{2}}{\underset{︸}{g_{n_{2}+1}=g_{n_{2}+1+{\left(n_{2}\right)}^{2}-1}=f_{n+1}^{\prime }}}.$$

If $F_{\sigma }=\left\{F_{\sigma_{n}}\right\}$ where $F_{\sigma_{n}}=g_{1}o\ldots og_{n}$ then $F_{\sigma }\in A\left(F\right)$. Now we claim that $h\left(F_{\sigma }\right)=\sum_{k=1}^{N}h\left(f_{k}\right).$

To prove this claim, take $c\left(n,i\right)=\sum_{j=1}^{n}\delta \left(g_{j}=f_{i}\right)$, where n∈N and 1≤i≤N. For every 1≤i≤N and n∈N, there exist A(n,i),B(n,i)∈N such that:

$$\begin{array}{c} g_{A(n,i)}=g_{A(n,i)+1}=\ldots =g_{B(n,i)}=f_{nN+i}^{\prime }. \end{array}$$

If $B\left(j,i\right)=n_{j}$, then $c_{\left(n_{j},i\right)}=c_{\left(B\left(j,i\right),i\right)}$. Thus

$$\begin{array}{c} \lim_{j\to \infty }\frac{c(n_{j},i)}{n_{j}}=\lim_{j\to \infty }\frac{c(B\left(j,i\right),i)}{n_{j}}\geq \lim_{j\to \infty }\frac{{\left(a_{ij}-1\right)}^{2}}{{\left(a_{ij}-1\right)}^{2}+a_{ij}}=1, \end{array}$$

where

$$\begin{array}{c} g_{a_{ij}}=g_{a_{ij}+1}=\ldots =g_{a_{ij}+{\left(a_{ij}-1\right)}^{2}}=f_{B(j,i)N+1}^{\prime }. \end{array}$$

Hence, ${lim sup}_{n}\frac{c(n,i)}{n}\geq 1$. Since for each 1≤i≤N,n∈N we have $\frac{c(n,i)}{n}\leq 1$, then ${lim sup}_{n}\frac{c(n,i)}{n}\leq 1$. Thus ${lim sup}_{n}\frac{c(n,i)}{n}=1$. Hence the claim is proved and $h\left(F_{\sigma }\right)=\sum_{i=1}^{N}h\left(f_{i}\right).$ ☐

The method of the proof of Theorem 7 yields that for any $x\in [a_{N},A_{N}]$ there is at least $F_{\sigma }\left(x\right)\in A\left(F\right)$ such that $h(F_{\sigma }\left(x\right))=x$, where $\min\left\{h\left(f_{k}\right)\right\}=a_{N}$ and $A_{N}=\sum_{k=1}^{N}h\left(f_{k}\right)$.

This fact and the above theorem motivate us to present the following definition.

**Definition** **6.** *Let* $F=(X,f_{1},\ldots ,f_{N})$ *be an* IFS*. Then we define the entropy of* F *by*

$$\begin{array}{c} h^{*}\left(F\right)=\sup\left\{h\left(F_{\sigma }\right):F_{\sigma }\in A\left(F\right)\right\}=\sum_{k=1}^{N}h\left(f_{k}\right). \end{array}$$

**Theorem** **8.** *If* $F=(X,f_{1},\ldots ,f_{N})$ *is topologically conjugate to* $K=(Y,k_{1},\ldots ,k_{N})$*, then* $h^{*}\left(F\right)=h^{*}\left(K\right).$

**Proof.**  Let ϕ:X⟶Y be a homeomorphism such that $\phi of_{i}=k_{i}o\phi$, for all i∈J. Then

$$\begin{array}{cc} h^{*}\left(F\right) & =\sum_{i=1}^{N}h\left(f_{i}\right)\\ & =\sum_{i=1}^{N}h\left(\phi^{-1}ok_{i}o\phi \right)\\ & =\sum_{i=1}^{N}h\left(k_{i}\right)\\ & =h^{*}\left(K\right). \end{array}$$

☐

**Theorem** **9.** *If* $\left(X,d_{1}\right)$ *and* $\left(Y,d_{2}\right)$ *are two compact metric spaces, and if* $F=(X,f_{1},\ldots ,f_{N})$*,* $G=(Y,g_{1},\ldots ,g_{M})$ *are two* IFS*, then*

$$\begin{array}{c} h^{*}\left(F\times G\right)=M\times h^{*}\left(F\right)+N\times h^{*}\left(G\right). \end{array}$$

**Proof.**  $$\begin{array}{cc} h^{*}\left(F\times G\right) & =\sum_{i,j}h\left(f_{i}\times g_{j}\right)\\ & =\sum_{i,j}\left(h\left(f_{i}\right)+h\left(g_{j}\right)\right)\\ & =\sum_{i,j}h\left(f_{i}\right)+\sum_{i,j}h\left(g_{j}\right)\\ & =M\times h^{*}\left(F\right)+N\times h^{*}\left(G\right). \end{array}$$

☐

**Theorem** **10.** *Let* $F=(X,f_{1},\ldots ,f_{N})$ *be an* IFS *on a compact topological space X then*

*(a)*  *If* $f_{1},\ldots ,f_{N}:X⟶X$ *are homeomorphisms, then* $h^{*}\left(F\right)=h^{*}\left(F^{-1}\right),$
*(b)*  $h^{*}\left(F^{m}\right)\geq mh^{*}\left(F\right)$*.*

**Proof.**  (a)
$\begin{array}{cc} h^{*}\left(F\right) & =\sum_{i=1}^{N}h\left(f_{i}\right)\\ & =\sum_{i=1}^{N}h\left(f_{i}^{-1}\right)\\ & =h^{*}\left(F^{-1}\right). \end{array}$ Theorem 7.3 of [7]
(b) Suppose that

$$\begin{array}{c} F^{m}:=\left(X,f_{I_{1}},...,f_{I_{N^{m}}}\right), \end{array}$$

where $f_{I_{i}}=f_{i_{m}}o\ldots of_{i_{1}}$ for $I_{i}=\left(i_{m},\ldots ,i_{1}\right)\in J^{m}$ and $1\leq i\leq N^{m}$, then
$\begin{array}{cc} h^{*}\left(F^{m}\right) & =\sum_{i=1}^{N^{m}}h\left(f_{I_{i}}\right)\\ & \geq \sum_{i=1}^{N}h\left(f_{i}^{m}\right)\\ & =\sum_{i=1}^{N}mh\left(f_{i}\right)\\ & =mh^{*}\left(F\right). \end{array}$ Theorem 7.10 of [7]☐

**Example** **4.** *Suppose X is the unit interval [0,1]. We consider the 2-ary expansion 0.* $x_{0}x_{1}x_{2}x_{3}\ldots$ *for* x∈[0,1] *and let* Σ:X⟶X *be the shift map, then* h(Σ)=log2 *(Theorem 7.12, [7]). In addition, if* Λ(x)=1−|1−2x| *is the tent map, then* h(Λ)=log2 *(Example 13, [25]). Thus for the* IFSF=(X,Σ(x),Λ(x)) *we have* $h^{*}\left(F\right)=log\;2+log\;2=log\;4$*.*

**Example** **5.** *In Example 2 with* F=(R,2x,3x) *we have* h(F)=log3*. By a similar method one can show that* h(f)=log2 *and* h(g)=log3 *where* f(x)=2x *and* g(x)=3x*. Thus* $h^{*}\left(F\right)=log\;3+log\;2>h\left(F\right).$

### 4.1. Non Wandering Sets and Topological Entropy of IFS

In this section we restrict ourself to IFSs on a compact manifold *M* which all of it’s functions are Axiom *A* diffeomorphisms.

A diffeomporphism f:M→M is an Axiom *A* diffeomorphism if

*(a)*  Ω(f) is a hyperbolic set and
*(b)*  the periodic points of *f* are dense in Ω(f), where Ω(f) is the set of non-wandering points of *f*.

We recall that, a point x∈M is called a non-wandering point if for each neighborhood *U* of *x* there is an n∈Z such that $f^{n}\left(U\right)\cap U\neq \emptyset$.

**Theorem** **11.** *Let* f∈Diff(M) *satisfies Axiom A then there is a neighborhood* $N_{f}$ *of f such that for every* $g\in N_{f}$ *have* h(f)≤h(g) *[26].*

Now we extend this theorem for iterated function systems.

**Theorem** **12.** *Let* $F=(X,f_{1},\ldots ,f_{N})$ *be an* IFS *such that for every* 1≤i≤N*,* $f_{i}\in Diff\left(M\right)$ *and it satisfies Axiom A. Then there is an* r>0 *such that for each* IFS*,* $G=(X,g_{1},\ldots ,g_{N})$ *with* $d(f_{i},g_{i})<r$ *for every* 1≤i≤N*, where* $d\left(f_{i},g_{i}\right)=\max\left\{d\left(f_{i}\left(x\right),g_{i}\left(x\right)\right):x\in M\right\}$*, we have* $h^{*}\left(F\right)\leq h^{*}\left(G\right).$

**Proof.**  In Theorem 11 there are $\left\{r_{f_{i}}\right\}$ with $d\left(f_{i},g\right)\leq r_{f_{i}}$ implies that $h\left(f_{i}\right)\leq h\left(g\right)$, 1≤i≤N. We choose $r=\min\{r_{f_{1}},\ldots ,r_{f_{N}}\}$. Then d(F,G)≤r, where $G=(X,g_{1},\ldots ,g_{N})$. So

$$\begin{array}{c} h^{*}\left(F\right)=\sum_{k=1}^{N}h\left(f_{k}\right)\leq \sum_{k=1}^{N}h\left(g_{k}\right)=h^{*}\left(G\right). \end{array}$$

☐

**Theorem** **13.** *Let* $F=(X,f_{1},\ldots ,f_{N})$ *be an* IFS *such that for every* 1≤i≤N*,* $f_{i}\in Diff\left(M\right)$ *and satisfies Axiom A and let σ be an arbitrary sequence in* ${\left\{1,2,\ldots ,N\right\}}^{N}$*. Then there is an* r>0 *such that for every* IFS*,* $G=(X,g_{1},\ldots ,g_{N})$ *with* $d(f_{i},g_{i})<r$ *for* 1≤i≤N *we have* $h\left(F_{\sigma }\right)\leq h\left(G_{\sigma }\right)$*.*

**Proof.**  We assume that $F_{\sigma }=\left\{F_{\sigma_{n}}\right\}\in A\left(F\right)$ and

$$\begin{array}{c} h\left(F_{\sigma }\right)=\sum_{k=1}^{N}b_{k}h\left(f_{k}\right), \end{array}$$

where $b_{k}={lim sup}_{n\to \infty }\frac{\sum_{i=1}^{n}\delta \left(f_{\sigma_{i}}=f_{k}\right)}{n}$. Consider the number r>0 and the IFS, $G=(X,g_{1},\ldots ,g_{N})$ as in the proof of Theorem 12. So $G_{\sigma }=\left\{G_{\sigma_{n}}\right\}\in A\left(G\right)$ with $h\left(G_{\sigma }\right)=\sum_{k=1}^{N}b_{k}^{\prime }h\left(g_{k}\right)$. Thus

$$\begin{array}{c} h\left(G_{\sigma }\right)=\sum_{k=1}^{N}b_{k}^{\prime }h\left(g_{k}\right), \end{array}$$

where $b_{k}^{\prime }={lim sup}_{n\to \infty }\frac{\sum_{i=1}^{n}\delta \left(g_{\sigma_{i}}=g_{k}\right)}{n}$, and $h(g_{k})$ is the topological entropy of the $g_{k}$. Since $h\left(f_{k}\right)\leq h\left(g_{k}\right)$, for every 1≤k≤N and $b_{k}=b_{k}^{\prime }$, then

$$\begin{array}{c} h\left(F_{\sigma }\right)=\sum_{k=1}^{N}b_{k}h\left(f_{k}\right)\leq \sum_{k=1}^{N}b_{k}^{\prime }h\left(g_{k}\right)=h\left(G_{\sigma }\right). \end{array}$$

☐

## 5. Conclusions

We consider topological entropy of iterated function systems from different ways, and we prove the essential properties of topological entropy for them. We conclude this paper with the following conjecture.

**Conjecture**: If $F=(X,f_{1},\ldots ,f_{N})$ then $h\left(F\right)\leq h^{*}\left(F\right).$
